# Photoelectric Multi-Signal Output Sensor Based on Two-Dimensional Covalent Organic Polymer Film Modified by Novel Aggregation-Induced Emission Probes

**DOI:** 10.3390/bios14060312

**Published:** 2024-06-18

**Authors:** Yaru Song, Guoling Wu, Enbing Zhang, Guangyuan Feng, Shengbin Lei, Lingli Wu

**Affiliations:** 1 Tianjin Key Laboratory of Molecular Optoelectronic Science, Department of Chemistry, School of Science & Collaborative Innovation Center of Chemical Science and Engineering (Tianjin), Tianjin University, Tianjin 300072, China; 2017210067@tju.edu.cn (Y.S.); wugl2020@163.com (G.W.); zhangenbing@tju.edu.cn (E.Z.); fengguangyuan@tju.edu.cn (G.F.); 2 Medical College, Northwest Minzu University, Lanzhou 730030, China

**Keywords:** fluorescence sensors, aggregation-induced emission (AIE), 2D materials

## Abstract

Optical sensors, especially fluorescence sensors, have been widely used because of their advantages in sensing, such as the high sensitivity, good selectivity, no radiation source, and easy operation. Here, we report an example of fluorescence sensing based on two-dimensional (2D) covalent organic polymers and highlight that the material can achieve a fast response and multi-signal output. This 2DP_TPAK+TAPB_-based sensor can quickly detect aromatic hydrocarbons and Fe^3+^ by the fluorescence signal or electrical resistance signal.

## 1. Introduction

Perception research plays a key role in the way we discover and learn more about the world [1,2]. Generally, a sensor is a device that helps us perceive and produce signal changes when interacting with an analyte. Among which, optical sensors, especially fluorescence sensors, have received extensive attention due to their advantages in terms of sensing, such as the high sensitivity, good selectivity, no radiation source, and easy operation [3,4]. A variety of fluorescent probes have been developed, such as metal organic frameworks, fluorescent polymers, quantum dots and organic fluorescent molecules. However, the relatively low sensitivity and complex synthesis process make them difficult to use for in situ detection, vapor phase sensing and recycling. The most important thing is that they can only output optical signals, and it is difficult to achieve multi-signal output, which is not conducive to practical application.

Two-dimensional (2D) covalent organic polymer films are covalently connected porous materials with π-conjugation and luminescence characteristics, and they are emerging candidates for the detection of various chemical substances [5,6,7,8,9,10]. First, it is easy to introduce specific sites into the conjugated chain, which could enhance the interfacial interaction and improve signaling activity with the target compound. Second, they have a rich pore structure, which provides a large interface for analyte interaction. Third, the inherent flexibility and thermal stability facilitate their application in flexible devices and harsh conditions. Finally, the formation of a thin film is convenient for their application in electrical devices to realize photoelectric multi-signal output. However, up to now, there is little report on the use of two-dimensional covalent organic polymers in fluorescence sensors and the realization of multi-signal output. Based on the above considerations, we report an example of fluorescence sensing based on two-dimensional covalent organic polymers and highlight that this material can achieve a fast response time and multi-signal output.

**Scheme 1:**
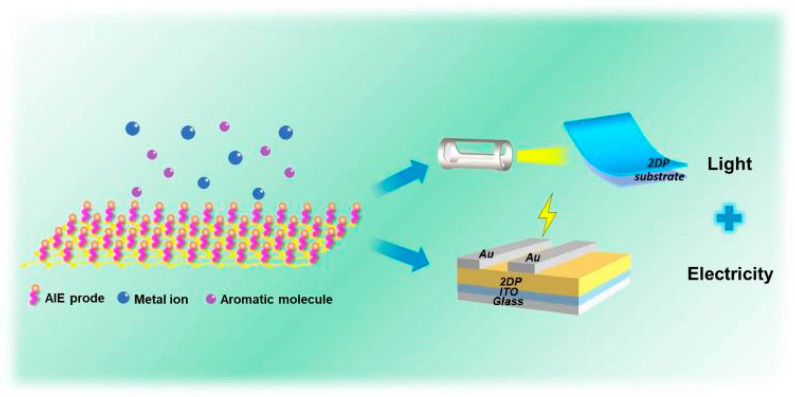
The strategy of constructing the multi-signal output sensor.

Here, we refer to the method of previous work used to prepare two-dimensional covalent organic polymer films (2DP_TPAK+TAPB_) via the Schiff base reaction by using the AIE-active 2,5-bis(3-(9H-carbazol-9-yl)propoxy)terephthalaldehyde (TPAK) molecule and 1,3,5-tris(4-aminophenyl)benzene (TAPB) molecule as building blocks [11]. Compared with other COF materials, 2DP_TPAK+TAPB_ can quickly detect electron-rich and electron-deficient aromatic hydrocarbons (10 s), showing the fluorescence-on and fluorescence-off sensing phenomena, respectively. In addition, it shows a good quenching response and selection to Fe^3+^ in solution. It is worth mentioning that, due to its inherent porosity, flexibility and thermal stability characteristics, it has good cycle stability (5 cycles), bending resistance (1000 times) and high temperature resistance (200 °C). Finally, we constructed a memristor with an Au/2DP_TPAK+TAPB_/ITO structure, which can detect electron-rich and electron-deficient aromatic vapors and Fe^3+^, and its HRS resistance also showed the resistance-on and resistance-off sensing phenomena, respectively. This work reports a fluorescent sensor based on two-dimensional covalent organic polymers and shows that it can achieve a fast response and photoelectric multi-signal output (Scheme 1).

## 2. Materials and Methods

**Figure 1:**
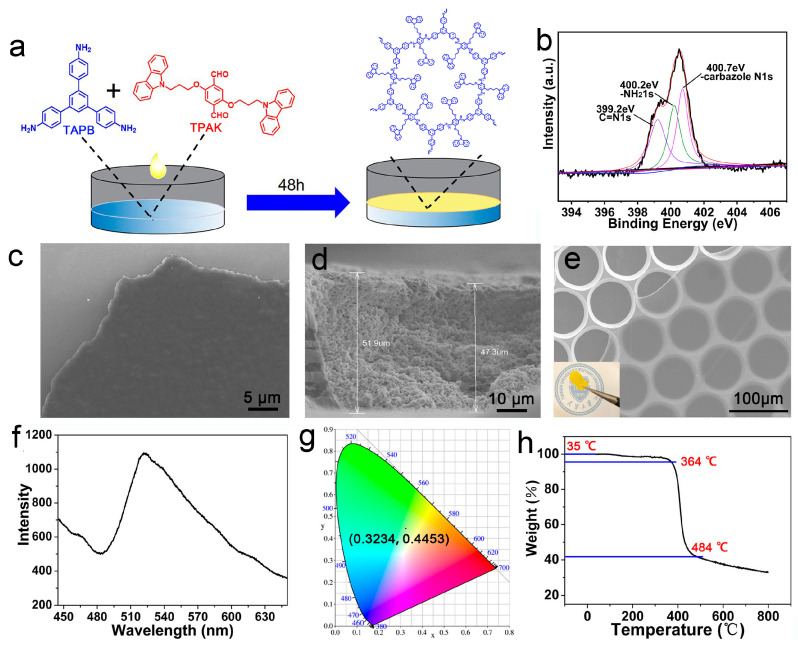
(**a**) The preparation diagram of 2DP_TPAK+TAPB_ on the gas–liquid interface. (**b**) XPS N 1s spectra of 2DP_TPAK+TAPB_. (**c**) SEM image of 2DP_TPAK+TAPB_ on Si/SiO_2_ substrate. (**d**) Cross-section SEM image of 2DP_TPAK+TAPB_. (**e**) SEM image of 2DP_TPAK+TAPB_ on a copper mesh substrate (the thickness of 2DP_TPAK+TAPB_ is 20.5 ± 0.5 nm, inset: thicker film of about 50 μm can be picked up directly with tweezers). (**f**) Fluorescence emission spectra of 2DP_TPAK+TAPB_ film (λ_ex_ = 365 nm). (**g**) Commission Internationale de L’Eclairage (CIE) coordinates of the emission color of 2DP_TPAK+TAPB_ film. (**h**) TGA data of 2DP_TPAK+TAPB_ film.

All of the chemicals were obtained from commercial sources and the synthetic diagram of 2DP_TPAK+TAPB_ is shown in Figure 1a.

### 2.1. Synthesis of Two-Dimensional Covalent Organic Polymer (2DP_TPAK+TAPB_)

First, 1,3,5-tris(4-aminophenyl)-benzene (TAPB, 7.0 mg, 0.02 mmol) and 2,5-bis(3-(9H-carbazol-9-yl)propoxy)terephthalaldehyde (TPAK, 17.4 mg, 0.03 mmol) were dissolved in 2 mL DMF and then 40 μL of acetic acid was added. Next, 200 μL of this mixture solution was diluted with 300 μL chlorobenzene and then carefully dropped on the surface of the 20 mL glycerin. After the bottle was allowed to stand at room temperature for 48 h, a layered two-dimensional polymer film was finally obtained at the solution/air interface.

### 2.2. Sensing Studies

For the solution-phase detection, the two-dimensional polymer film was placed in a certain concentration (0 to 1.0 × 10^−2^ M) of metal ion aqueous solution for a specified period of time (0 to 600 s). It was then removed, the excess water was soaked up with paper, and it was installed on the sample holder of the fluorescence spectrometer without delay. The emission spectrum was measured and recorded.

For the gas-phase sensing, a solid (100 mg) or liquid (1 mL) of explosive molecules or volatile organic compound (VOC) molecules was placed in a sealed vial at room temperature for about 7 d to ensure the saturation of the aromatic hydrocarbon vapor in the vial, and then the two-dimensional polymer film was placed in the vial for a specified period of time to be exposed to the vapor, then removed, mounted without any delay on the sample holder of the spectrofluorometer, and the emission spectrum was recorded. The raw emission spectra of the sample 2D polymer film were collected before placing the 2D polymer film into the bottle containing aromatic hydrocarbons.

In the repeatability (cycling) tests, for the gas-phase sensing, 2D polymer films were exposed to aromatic hydrocarbon vapors, measured with a spectrofluorometer, evacuated at 25 °C for about 6 h to remove the adsorbed aromatics, placed in air for about 1 h in the dark, and then the next test was carried out. For the solution-phase detection, the 2D polymer films were in an aqueous solution of metal ions for a certain time, measured with a fluorescence spectrometer, and washed repeatedly with deionized water about 10 times to remove the adsorbed metal ions, and then the study proceeded to the next test.

### 2.3. Thermogravimetric Analysis (TGA) Test

In order to remove the unreacted monomers from the synthesized two-dimensional polymer film, the film was gently transferred to a DMF solvent, soaked for 30 min, and collected. The test film samples were dried in a vacuum-drying oven at 80 °C for 12 h. During the test, the temperature range was 35–750 °C, the heating rate was 10 °C/min, and the nitrogen flow rate was 20 mL/min.

### 2.4. Device Fabrication of Memristor Based on 2DP_TPAK+TAPB_ Film

The indium-tin-oxide (ITO) glass substrates were sequentially cleaned with toluene, acetone, ethyl alcohol, isopropanol and deionized water. The synthesized 2DP_TPAK+TAPB_ films were first transferred onto the surface of DMF to remove any unreacted precursors. Then, the 2DP_TPAK+TAPB_ films were picked up by the ITO glass substrates. The top electrode of Au (50 nm) was deposited on the 2DP_TPAK+TAPB_ with the help of a copper mask. The memory characteristics of the devices were measured using a probe station at room temperature.

### 2.5. Characterization

Scanning electron microscope (SEM) images were taken using a Hitachi SEM SU8010 field emission scanning electron microscope (FESEM). The fluorescence spectroscopy was carried out using an F-7000 fluorescence spectrometer. The X-ray photoelectron spectroscopy (XPS) spectrum was tested with a Thermo Fisher Scientific ESCALAB 250Xi. The electric properties were measured using a micromanipulator 6150 probe station connected to a Keithley 4200-SCS.

## 3. Results and Discussion

### 3.1. Characterization of the 2DP_TPAK+TAPB_

First, the TPAK monomer was synthesized with reference to previous work [11]. It had obvious aggregation-induced emission (AIE) behavior (Figure S1). Then, the imine-based two-dimensional covalent organic polymer films (2DP_TPAK+TAPB_) were synthesized by the reaction of TAPB with TPAK at the gas–liquid interface (Figure 1a). The obtained 2DP films were transferred to an Si/SiO_2_ substrate for morphology characterization using a scanning electron microscope (SEM). Under scanning electron microscopy, the film is relatively smooth and uniform (Figure 1c). The thickness of the 2DP films can be easily adjusted between several nanometer and about 50 μm by tunning the amount of solution deposited. As shown in Figure 1d, the SEM images show the porous structure of a 2DP_TPAK+TAPB_ film with thickness of about 49.6 ± 2.3 μm. The XPS spectra were used to further characterize the chemical structure of the 2DP films and confirmed the successful formation of the -C=N bonds. As shown in Figure 1b, the N 1s spectrum of 2DP_TPAK+TAPB_ can be deconvoluted into three components, the lower binding energy component centered at 399.2 eV can be attributed to the imine bonds, the one at 400.2 eV corresponds to the unreacted -NH_2_ of TAPB and the one at 400.7 eV is from the carbazole-N, which clearly confirms the formation of imine bonds and the incomplete reaction of the precursors, which is consistent with our previous results [11].

We further explored the optical properties and stability of the 2DP_TPAK+TAPB_ film. The fluorescence emission maxima of the 2DP_TPAK+TAPB_ film is 522 nm (Figure 1f). The observed emission color of the 2DP_TPAK+TAPB_ film is yellow on the Commission Internationale de L’Eclairage (CIE) chromaticity diagram, with the coordinates (0.3234, 0.4453) (Figure 1g). Furthermore, due to its covalently bonded structure, the 2DP_TPAK+TAPB_ film has good thermal stability and flexibility. TGA data show that the 2DP_TPAK+TAPB_ film is stable up to 360 °C, while the weight loss at 35–364 °C (3.5%) can be attributed to the loss of adsorbate and unreacted monomers. The rapid weight loss at 364–484 °C can be attributed to carbonization (Figure 1h). The scanning electron microscope (SEM) image (Figure 1e) also shows that the film as thin as 20.5 ± 0.5 nm still has good self-supporting characteristics. In addition, the thicker film of about 50 μm can be picked up directly with tweezers (inset of Figure 1e). The excellent thermal stability and mechanical properties of the 2DP_TPAK+TAPB_ film make fluorescence sensors based on it have broad prospects in practical applications.

### 3.2. Performance of Sensor Based on the 2DP_TPAK+TAPB_

As the main source of air pollution, volatile organic compounds (VOCs) pose various threats to homeland security and human health. VOCs are the main source of secondary pollutants such as photochemical smog and haze, tropospheric ozone and peroxyacetyl nitrate in urban areas. There is no doubt that high concentrations of and/or long-term exposure to VOCs may increase the risk of acute or chronic poisoning, headaches, lung cancer, and central nervous system damage [12]. In addition, due to the release of VOCs in the chemical industry, cosmetics, building materials, etc., they exist in indoor/outdoor air. Therefore, effective detection of VOCs is essential to the protection of the environment and health [12]. In addition, nitro aromatic explosives, such as 2,4-dinitrophenol (DNP), are the main components of military explosives and landmines. They have mutagenic effects that induce acute health problems and are the main source of environmental pollution [13,14]. Although many materials for detecting VOCs and explosive in solution have been widely developed, the fluorescence detection of trace solvent vapor is still challenging due to its low vapor pressure at room temperature. Therefore, the detection of VOCs in the gas phase at room temperature is much more difficult than in the liquid form. 

**Figure 2:**
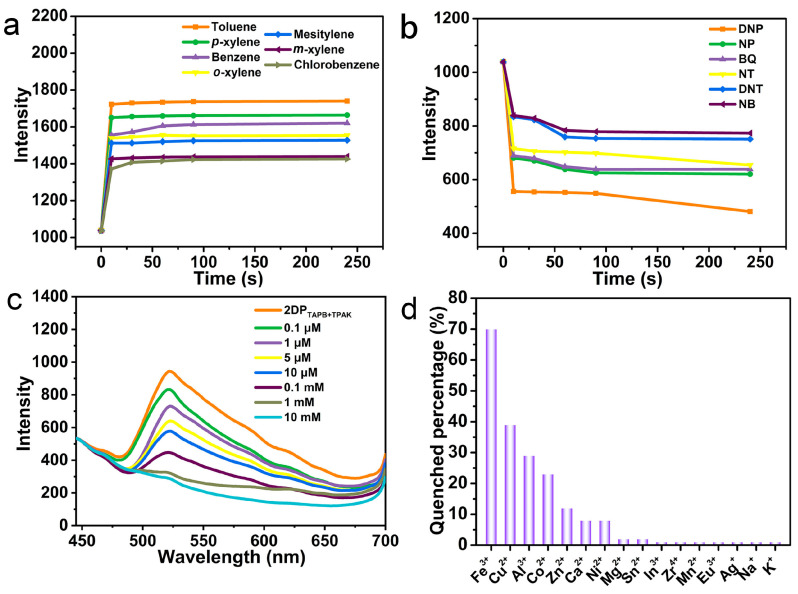
(**a**) Fluorescence enhancement of 2DP_TPAK+TAPB_ upon exposure to vapors of toluene, *p*-xylene, benzene, *o*-xylene, mesitylene, *m*-xylene, and chlorobenzene. (**b**) Fluorescence quenching of 2DP_TPAK+TAPB_ upon exposure to vapors of 2,4-dinitrophenol (DNP), p-nitrophenol (NP), 4-nitrotoluene (NT), 2,4-dinitrotoluene (DNT), 1,4-benzoquinone (BQ) and nitrobenzene (NB). (**c**) Luminescence spectra of 2DP_TPAK+TAPB_ after immersion in different concentrations of Fe^3+^ (0 to 1.0 × 10^−2^ M, soaking time = 60 s). (**d**) The luminescence quenching percentage of 2DP_TPAK+TAPB_ after immersion in various metal ions. [metal ion] = 1.0 × 10^−2^ M, Soaking time = 60 s.

We studied the fluorescence sensing behavior of 2DP_TPAK+TAPB_ for VOCs and nitrophenol explosives. Their molecular structures are shown in Figure S2. Sensing experiments were conducted by exposing samples of 2DP_TPAK+TAPB_ to arene vapors for specific periods of time at 25 °C and then performing fluorescence spectroscopy (Figures S3 and S4). The results showed that 2DP_TPAK+TAPB_ is quite sensitive to arenes. For example, upon exposure to the vapor of toluene, benzene, mesitylene, and chlorobenzene for only 10 s, the fluorescence intensity of 2DP_TPAK+TAPB_ increased by 64%, 50%, 46%, 32% and gradually reached saturation (240 s) (Figure 2a). It is obvious that the toluene vapor causes the greatest enhancement. It is worth noting that, after exposure to xylene isomer vapor for 10 s, the response of the fluorescence intensity of 2DP_TPAK+TAPB_ is also different (*p*-xylene: 58%, *o*-xylene: 47%, *m*-xylene: 34%), indicating that 2DP_TPAK+TAPB_ also has a certain ability to distinguish xylene isomers. Just like electron-deficient aromatic hydrocarbons, the microporous framework of 2DP_TPAK+TAPB_ can be approached by electron-deficient aromatic hydrocarbons. However, it is interesting that, contrary to the fluorescence enhancement caused by electron-rich aromatics, the presence of electron-deficient aromatics quenches the fluorescence intensity of 2DP_TPAK+TAPB_. For example, after exposure to the vapor of 2,4-dinitrophenol (DNP), p-nitrophenol (NP), 1,4-benzoquinone (BQ), 4-nitrotoluene (NT), 2,4-dinitrotoluene (DNT) and nitrobenzene (NB) for only 10 s, the fluorescence intensity of 2DP_TPAK+TAPB_ was quenched by 46%, 35%, 34%, 31%, 20%, 19% and gradually reached saturation (240 s) (Figure 2b). It can be clearly seen that 2,4-dinitrophenol (DNP) vapor is the most obvious quenching reagent. The rapid response and rapid saturation of the fluorescence intensity indicate that the porous network is extremely sensitive to these aromatics.

Fe^3+^ ions affect various important cell functions, such as muscle function, hemoglobin formation and brain function. Excess and deficiency beyond the normal allowable limits can cause various health hazards [12]. Therefore, the selective detection of Fe^3+^ ions is very important for human health. Here, we studied the fluorescence-sensing behavior of 2DP_TPAK+TAPB_ in relation to 16 kinds of metal ions. Figure 2c studies the concentration-dependent fluorescence-quenching experiment of 2DP_TPAK+TAPB_ on Fe^3+^. It can be seen that with the increase in the Fe^3+^ ion concentration, the fluorescence intensity of 2DP_TPAK+TAPB_ gradually decreases. When the Fe^3+^ ion concentration is only 1.0 × 10^−4^ M, the fluorescence intensity is quenched by more than 50%. Figure S5 shows the linear Stern–Wolmer curve, the linear range is 0–1000 μM, and the calculated quenching constant (K_SV_) and LOD are 2.7 × 10^5^ M^−1^ and 4.2 × 10^−7^ M, respectively. Figure S6a shows the time-dependent fluorescence-quenching experiment of 2DP_TPAK+TAPB_ on Fe^3+^. When the 2DP_TPAK+TAPB_ film is soaked in Fe^3+^ solution of a 1.0 × 10^−4^ M concentration for about 1 min, its fluorescence intensity is quenched by more than half. In addition, the selectivity and sensitivity are important criteria for sensing. As shown in Figure 2d, the quenching degree of 2DP_TPAK+TAPB_ by the Fe^3+^ ions is much higher than that of the other metal ions, and the selectivity is very good. Figure S6b shows that in presence of other metal ions, the quenching degree of 2DP_TPAK+TAPB_ on Fe^3+^ is almost unchanged. The results show that the addition of other metal ions hardly interferes with the selective sensing of Fe^3+^ ions by 2DP_TPAK+TAPB_. 

The above-mentioned advantages in terms of the luminescent properties of 2DP_TPAK+TAPB_ have provided a powerful motivation for evaluating whether it is suitable for actual water samples containing Fe^3+^ ions. The water from the Haihe River in Tianjin was collected and the sensing experiments were carried out. As shown in Table S1, the Fe^3+^ ion concentration in the water samples can be accurately determined, and the recovery rate is high (98.72–106.89%), which shows that it is suitable for quantitative monitoring of Fe^3+^ in environmental water.

**Figure 3:**
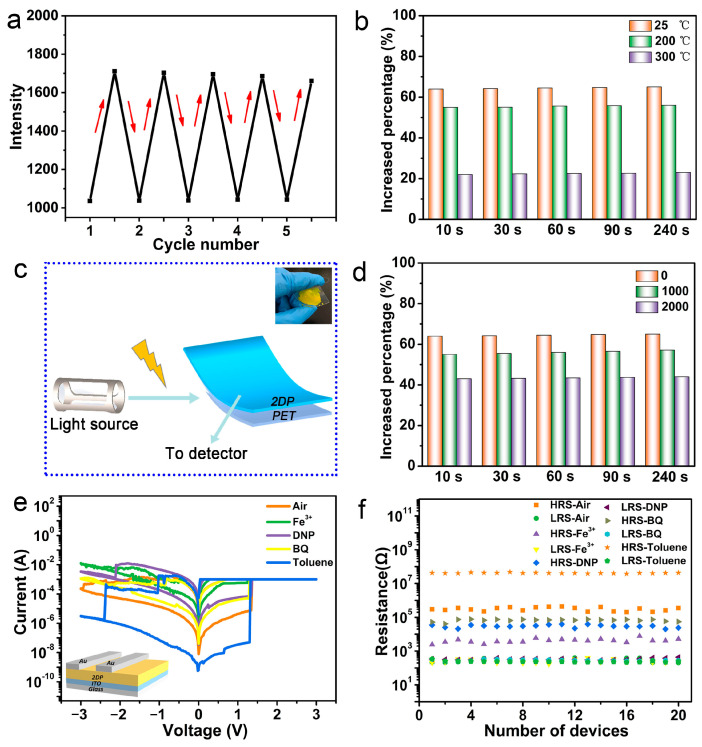
(**a**) Cycling tests of 2DP_TPAK+TAPB_ upon exposure to vapors of toluene. (**b**) High-temperature test of 2DP_TPAK+TAPB_ upon exposure to vapors of toluene. (**c**) Schematic diagram of flexible fluorescent sensor (inset: actual photo of 2DP_TPAK+TAPB_/PET). (**d**) Bending resistance test of 2DP_TPAK+TAPB_ upon exposure to vapors of toluene. (**e**) I–V characteristics of the Au/2DP_TPAK+TAPB_/ITO device under different conditions. (**f**) HRS and LRS resistance distribution among different Au/2DP_TPAK+TAPB_/ITO devices (20) under different conditions (read at 0.1 V). The Au/2DP_TPAK+TAPB_/ITO device in air, DNP, BQ, and toluene atmosphere for 30 min; the 2DP_TPAK+TAPB_ film immersed in Fe^3+^ solution, [Fe^3+^] = 1.0 × 10^−2^ M, Soaking time = 60 s.

We continued to study the reversibility, repeatability and stability of the fluorescence sensors based on 2DP_TPAK+TAPB_. Here, 2DP_TPAK+TAPB_ can be used repeatedly in the fluorescence-on and -off induction of aromatic hydrocarbons. For example, after exposure to toluene vapor for 10 s, 2DP_TPAK+TAPB_ showed almost the same degree of fluorescence enhancement after each cycle, and when the toluene vapor was removed, the fluorescence intensity returned to a similar level. Therefore, 2DP_TPAK+TAPB_ maintains the high sensitivity and fast response speed (Figure 3a). In addition, for other VOCs such as 1,4-benzoquinone (BQ), the fluorescence sensing of 2DP_TPAK+TAPB_ also shows good repeatability and stability (Figure S7a). Due to the interweaving nature of the network skeleton, 2DP_TPAK+TAPB_ can be reused without a significant decrease in sensitivity and responsiveness for VOCs. However, for Fe^3+^ ions in the solution, the fluorescence intensity cannot be restored to a similar level because the Fe^3+^ ions cannot be completely removed after each post-treatment, but it can still be reused for about five cycles (Figure S8a). So far, most fluorescent sensors can only work at room temperature and show limited heat resistance [10,15,16,17,18,19,20]. However, in the current work, we speculate that the high thermal stability of 2DP_TPAK+TAPB_ film makes it an ideal material for manufacturing sensors with high thermal stability. In order to further evaluate the thermal stability, we heated the about 50 μm thick 2DP_TPAK+TAPB_ film in a cubic furnace protected by argon at a high temperature for 2 h. The fluorescent-sensing characteristics after heating are shown in Figure 3b, Figure S7b and Figure S8b. Even after annealing at 200 °C, the fluorescence sensor based on 2DP_TPAK+TAPB_ has the same degree of response to toluene, BQ, and Fe^3+^ as the results at room temperature. The high thermal stability is attributed to the strong covalent bonds in the 2DP_TPAK+TAPB_ film. This will be more conducive to the application of sensors in actual production, such as the aerospace, geothermal, oil and gas industries. In addition, bending resistance is also an important criterion for constructing flexible sensing equipment. Here, we show a fluorescent sensor on a flexible substrate (PET) (Figure 3c, Figure S7c and Figure S8c). Even after 1000 bending cycles, the 2DP_TPAK+TAPB_-based fluorescence sensor has the same degree of response to toluene, BQ, and Fe^3+^ (Figure 3d, Figure S7d and Figure S8d). Therefore, the 2DP_TPAK+TAPB_ film with good flexibility and high thermal stability is very attractive for constructing sensors that are more conducive to practical applications in harsh environments.

Realizing the multi-signal output of the sensor has always been one of the pursuits of scientists. However, so far, there are few reports on the multi-signal output of fluorescence sensors. Therefore, we expect to realize the multi-signal output of the sensor based on the two-dimensional covalent organic polymer film. Inspired by previous work [11], this material can be applied to memristors with a vertical structure Au/2DP_TPAK+TAPB_/ITO. Here, we built the Au/2DP_TPAK+TAPB_/ITO device, and then we placed the device in a different steam environment and took it out after about 30 min to test its I–V characteristics. It is worth pointing out that for Fe^3+^ detection, we directly prepared the device using the film soaked in Fe^3+^ solution and then tested it. The results are shown in Figure 3e, and compared with the test results in the air, for electron-rich toluene, the high resistance state (HRS) current decreases and the HRS resistance increases. For electron-deficient BQ, DNP, Fe^3+^, the HRS current rises and the HRS resistance decreases. To further prove the result, we counted about 20 devices, and the result is shown in Figure 3f. The average value of the HRS resistance of the pristine device in the air is 3.13 × 10^5^ Ω, and the average value of the HRS resistance of the device in toluene vapor is 4.18 × 10^7^ Ω, which is about 2 orders of magnitude different from the result in the air. The average values of the HRS resistances of the devices in BQ, DNP, and Fe^3+^ are 6.75 × 10^4^ Ω, 4.26 × 10^3^ Ω, 3.00 × 10^4^ Ω, which are 1–2 orders of magnitude different from the results in air.

### 3.3. Mechanism of Sensor Based on the 2DP_TPAK+TAPB_

**Figure 4:**
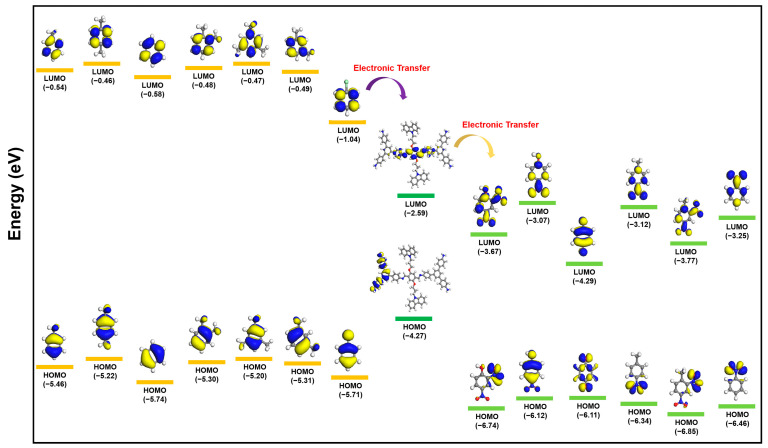
Schematic representation of electron transfer. The energy levels were simulated theoretically by Materials Studio 2019 software (from left to right: toluene, *p*-xylene, benzene, *o*-xylene, mesitylene, *m*-xylene, chlorobenzene, 2DP_TPAK+TAPB_, 2,4-dinitrophenol (DNP), p-nitrophenol (NP), 1,4-benzoquinone (BQ), 4-nitrotoluene (NT), 2,4-dinitrotoluene (DNT) and nitrobenzene (NB)).

We further studied the possible sensing mechanism of VOCs, nitro aromatic explosives and Fe^3+^ ion-quenching luminescence. According to our previous work [11], the energy levels of the highest occupied molecular orbital (HOMO) and lowest unoccupied molecular orbital (LUMO) of the 2DP_TPAK+TAPB_ film were determined to be −5.51 eV and −3.03 eV, respectively, which are close to the theoretical simulation value (HOMO/LUMO: −4.27 eV/−2.59 eV). The bottom of the conduction band of 2DP_TPAK+TAPB_ is higher than the lowest unoccupied molecular orbital (LUMO) of the electron-deficient aromatics, which drives electron transfer from the 2DP_TPAK+TAPB_ to the electron-deficient aromatics and causes fluorescence quenching, as observed with other conjugated polymers (Figure 4) [21,22,23,24,25]. While the LUMO of the electron-rich aromatic hydrocarbon is higher than the conduction band of 2DP_TPAK+TAPB_, under illumination the electrons transfer from the aromatic hydrocarbon to the conduction band of 2DP_TPAK+TAPB_, thereby enhancing the fluorescence intensity (Figure 4) [21,22,23,24,25]. The fluorescence-on and -off mechanisms of 2DP_TPAK+TAPB_ allow it to clearly distinguish electron-rich and electron-deficient aromatics. In addition, it is well known that Fe^3+^ has an empty d orbital and has a chelating ability with a special N atom compared with other metal ions [26,27]. For the electron-rich 2DP_TPAK+TAPB_, the electron-deficient metal cation can quench the fluorescence of 2DP_TPAK+TAPB_ through electron transfer. Two factors affect electron transport: standard electrode potential and metal ion diameter. Fe^3+^ has a high standard electrode potential (0.77 V) and small diameter (1.1 Å) [28]. Therefore, 2DP_TPAK+TAPB_ has the highest response to Fe^3+^. 

## 4. Conclusions

In summary, we report a new strategy for constructing molecular detection systems using two-dimensional covalent organic polymer films. The 2DP_TPAK+TAPB_-based fluorescence sensor can detect electron-rich and electron-deficient aromatic hydrocarbons quickly (10 s), showing the fluorescence-on and fluorescence-off sensing phenomena, respectively. In addition, it shows a good quenching response and selection for Fe^3+^ ions in solution. It is worth mentioning that due to its inherent porosity, flexibility and thermal stability characteristics, 2DP_TPAK+TAPB_ has good cycle stability (5 cycles), bending resistance (1000 times) and high temperature resistance (200 °C). Importantly, we constructed a memristor-based sensor with an Au/2DP_TPAK+TAPB_/ITO structure, which can detect electron-rich and electron-deficient aromatic vapors and Fe^3+^, and its HRS resistance also showed the resistance-on and resistance-off sensing phenomena, respectively. Compared with other COF materials, 2DP_TPAK+TAPB_ has certain advantages in the detection experiment of VOCs, nitro aromatic explosives and metal ions in terms of the detection speed, stability (high temperature resistance, bending resistance), actual sample detection and multi-signal output (Tables S2 and S3). The electron-rich and electron-deficient aromatic vapors and Fe^3+^ enhance or quench the fluorescence of 2DP_TPAK+TAPB_ through the electron transfer mechanism. This work reports an example of fluorescence sensing based on two-dimensional covalent organic polymers and the realized photoelectric multi-signal output. Although its performance and its further application in actual production still need to be improved, as a proof of concept, we provide a new method and material to design a multi-signal output sensor with excellent performance.

## Data Availability

Data are contained within the article and Supplementary Materials.

## Supplementary Materials

The following supporting information can be downloaded at https://www.mdpi.com/article/10.3390/bios14060312/s1 [29,30,31,32,33,34,35], Figure S1: The AIE behavior of TPAK; Figure S2: The molecular structure of VOCs and nitrophenol explosives; Figure S3: Fluorescence spectra of 2DP_TPAK+TAPB_ to the electron-rich aromatic hydrocarbons; Figure S4: Fluorescence spectra of 2DP_TPAK+TAPB_ to the electron-deficient aromatic hydrocarbons; Figure S5: Plot of the PL-quenching efficiency (I_0_/I) as a function of the Fe^3+^ concentration; Figure S6: Concentration-dependent fluorescence-quenching experiment of 2DP_TPAK+TAPB_ on Fe^3+^ and its selectivity; Figure S7: The reversibility, repeatability and stability of the 2DP_TPAK+TAPB_ fluorescence sensors to the electron-deficient aromatic hydrocarbons (BQ); Figure S8: The reversibility, repeatability and stability of the 2DP_TPAK+TAPB_ fluorescence sensors to the metal ion (Fe^3^+); Table S1: The recovery rate experiment of the 2DP_TPAK+TAPB_ fluorescence sensors; Table S2: The performance of 2DP_TAPB+TPAK_ in the detection experiment of VOCs/explosive; Table S3: The performance of 2DP_TAPB+TPAK_ in the detection experiment of metal ions.
