# Supplementary material for: Photoelectric Multi-Signal Output Sensor Based on Two-Dimensional Covalent Organic Polymer Film Modified by Novel Aggregation-Induced Emission Probes
Source: Biosensors (Basel). 2024 Jun 18;14(6):312. doi: 10.3390/bios14060312 (PMC11202238; doi:10.3390/bios14060312)
Supplement: Supplementary file 1 [file biosensors-14-00312-s001.zip › biosensors-2990761-supplementary.pdf]

biosensors

MDPI

supplementary

Photoelectric Multi-Signal Output Sensor Based on Two-Dimensional Covalent Organic Polymer Film Modified by Novel Aggregation-Induced Emission Probes

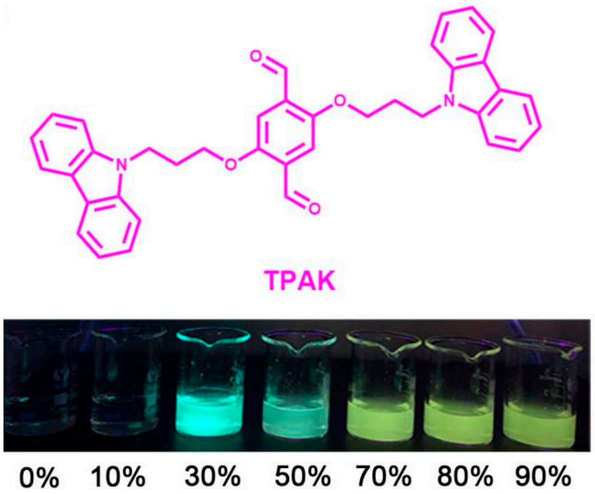

Figure S1. The AIE behavior of TPAK: photograph of TPAK in DMF/H_2O mixtures with different fw of H_2O under UV light (365 nm).

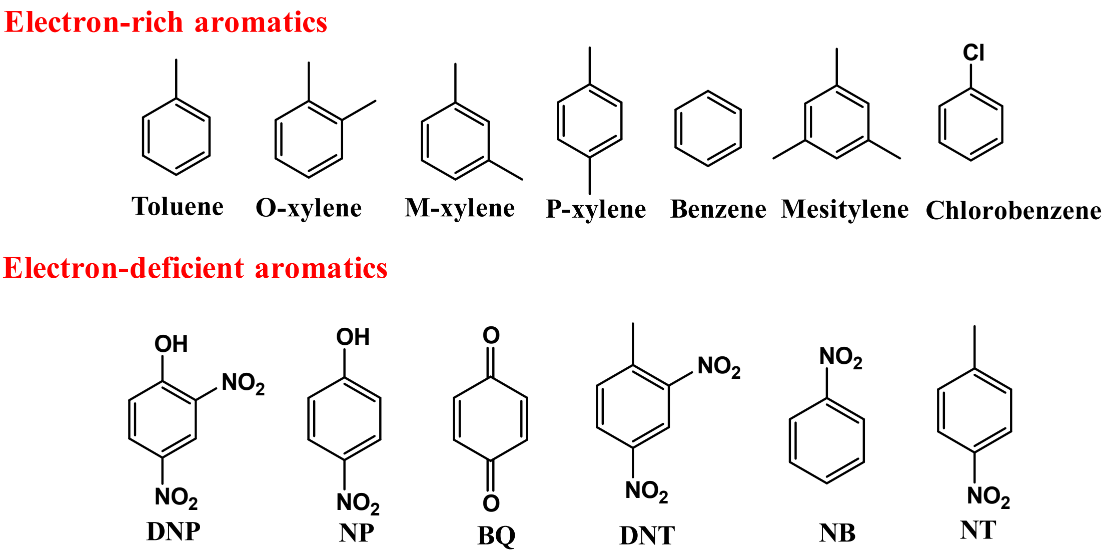

Figure S2. The molecular structure of VOCs and nitrophenol explosives used in this article.

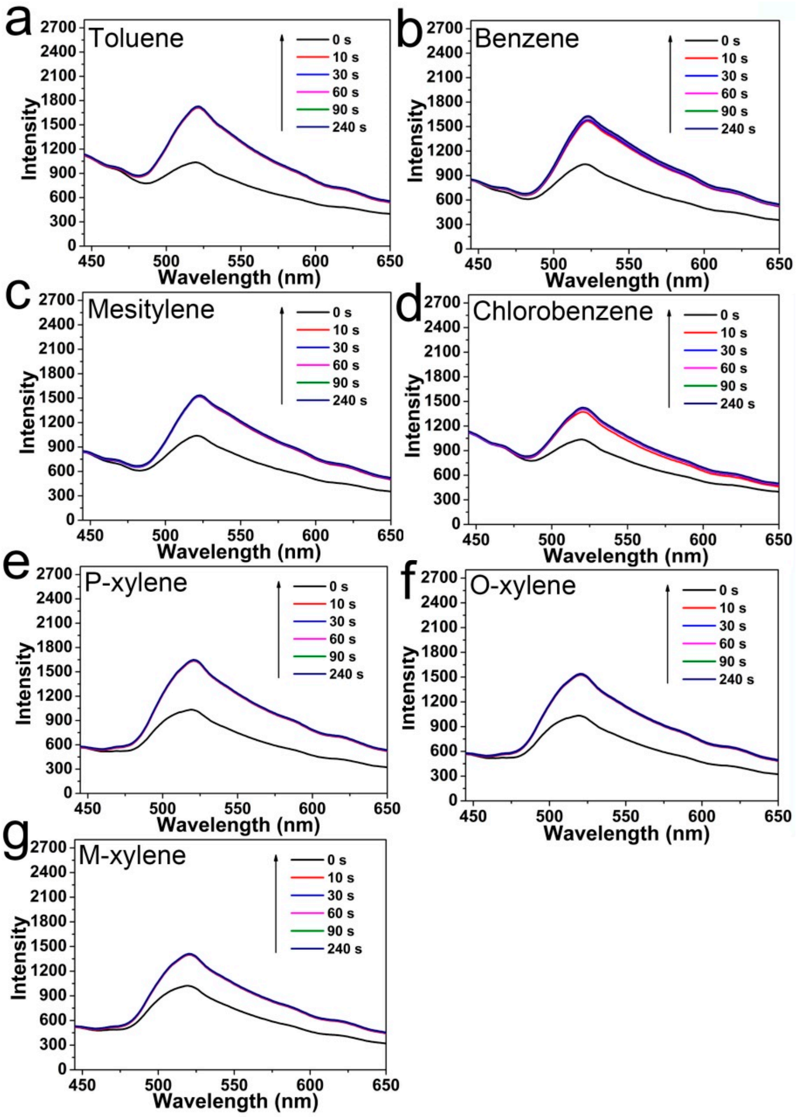

Figure S3. Fluorescence spectral changes of 2DP$_{TPAK+TAPB}$ film upon exposure to the vapors of electron-rich aromatic hydrocarbons.

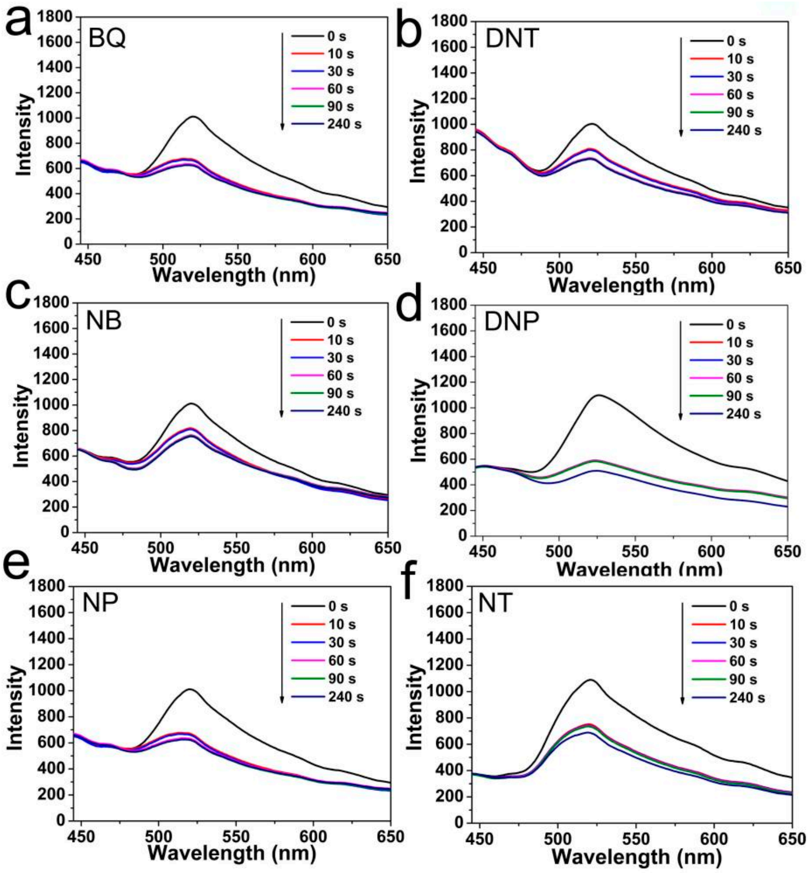

Figure S4. Fluorescence spectral changes of 2DPTPAK+TAPB film upon exposure to the vapors of electron-deficient aromatic hydrocarbons and nitrophenol explosives.

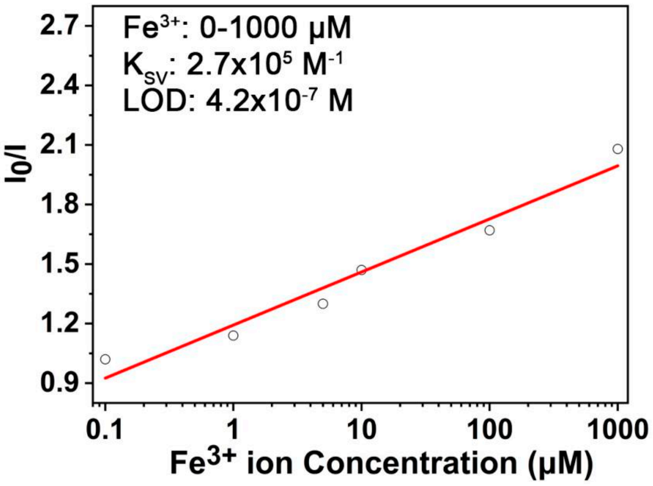

Figure S5. Plot of PL quenching efficiency (I_0/I) as a function of Fe^{3+} concentration (0-1000 μM). The detection limit = 3 × S.D./k, where k is the slope of the curve equation, and S.D. Represents the standard deviation for the 2DPTPAK+TAPB film intensity in the absence of Fe^{3+}.

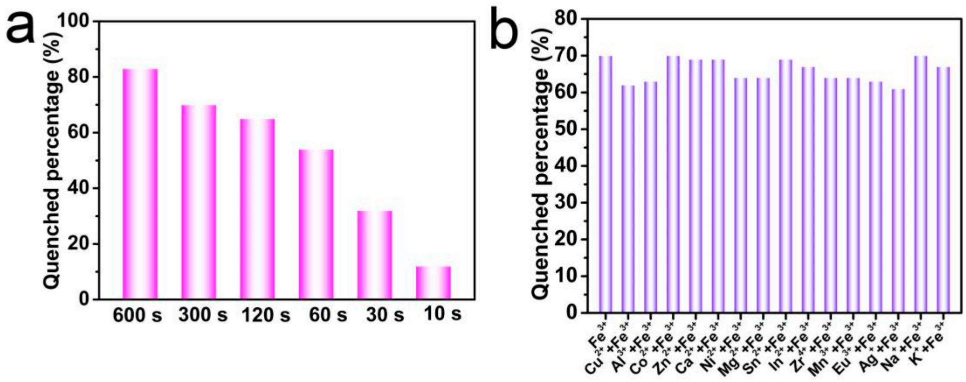

Figure S6. a. Luminescence intensity of $2DP_{TPAK+TAPB}$ at different time after immersed in 1.0×10^{-4} M Fe^{3+}. **b.** Luminescence intensity of $2DP_{TPAK+TAPB}$ in the presence of Fe^{3+} ions and other metal ions. $[Fe^{3+}] = 1.0\times10^{-2}$ M, [metal ion] = 1.0×10^{-2} M, Soaking time = 60 s.

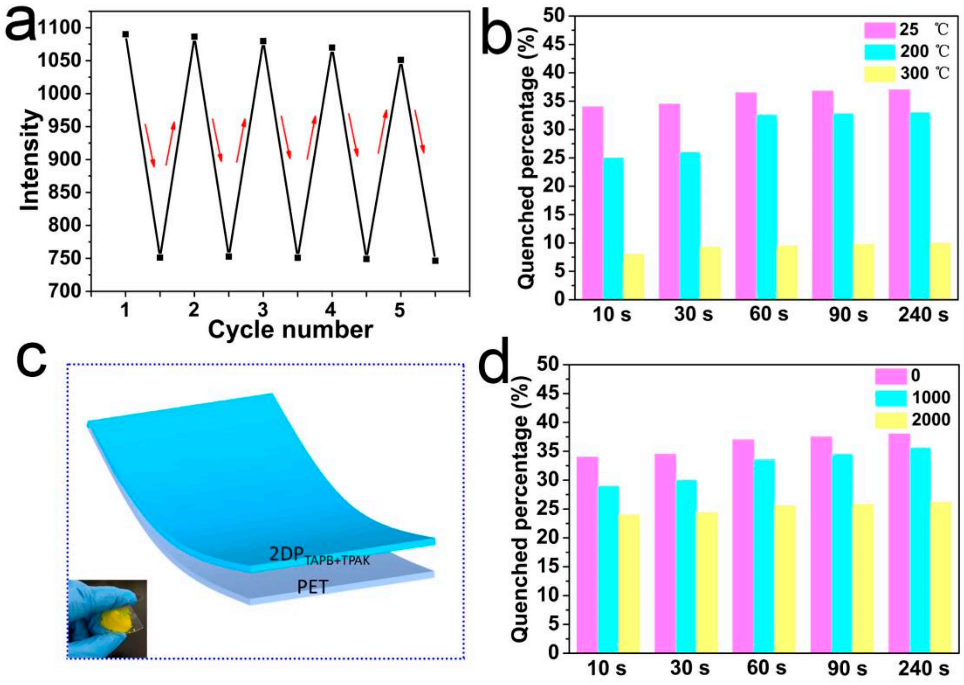

Figure S7. a. Cycling tests of $2DP_{TPAK+TAPB}$ upon exposure to vapors of BQ. **b.** High temperature test of $2DP_{TPAK+TAPB}$ upon exposure to vapors of BQ. **c.** Schematic diagram of flexible fluorescent sensor (inset: actual photo of $2DP_{TAPB+TPAK}$/PET). **d.** Bending resistance test of $2DP_{TPAK+TAPB}$ upon exposure to vapors of BQ.

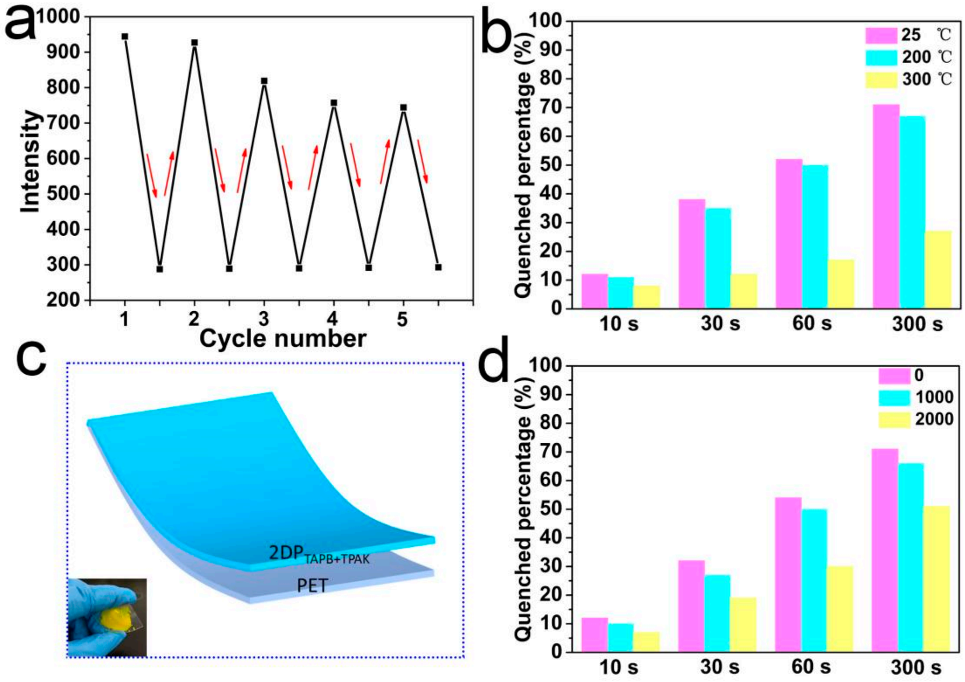

Figure S8. a. Cycling tests of $2DP_{TPAK+TAPB}$ after immersed in Fe^{3+}. **b.** High temperature test of $2DP_{TPAK+TAPB}$ after immersed in Fe^{3+}. **c.** Schematic diagram of flexible fluorescent sensor (inset:.actual photo of $2DP_{TAPB+TPAK}$/PET). **d.** Bending resistance test of $2DP_{TPAK+TAPB}$ after immersed in Fe^{3+}. $[Fe^{3+}] = 1.0\times10^{-4}$ M, soaking time = 60 s.

Table S1. Results for the determination of Fe^{3+} in Haihe River by the standard addition method. All concentrations were expressed as mean of three measurements.

Sample	Added (μM)	Found (μM)	Recovery (%)	RSD (%)
1	**10**	**10.689**	**106.89**	**2.55**
2	**8**	**8.433**	**105.41**	**2.28**
3	**6**	**5.923**	**98.72**	**5.16**

Table S2. The performance of 2DP$_{TAPB+TPAK}$ in the detection experiment of VOCs/explosive.

Material	Status	VOC/Explosive	Detected object status	Responding speed	Responding degree	Cycles	High temperature resistance	Bending resistance	Literature
Py-Azine COF	Powder	TNP	Gas	-	69%	-	-	-	1
TfpBDH-CONs	Nanosheets	TNP	Solution	60 min	63%	-	-	-	2
3D-Py-COF	Powder	PA	Solution	-	75%	-	-	-	3
PI-CONs	Nanosheets	TNP	Solution	2 min	64%	-	-	-	4
COF-BABD-BZ	Powder	TNP	Solution	Few minutes	92.3%	-	-	-	5
HS	Powder	TNP	Gas	2 h	99%	-	-	-	6
NUS-20	Powder	Nitrobenzene/ Toluene	Gas	15 s	83%/152%	10	-	-	7
NUS-24	Nanosheets	Nitrobenzene/ 1,4-isopropylbenzene	Solution	20 min	87%/16%	-	-	-	8
2DP$_{TPAK+TAPB}$	Film	Toluene/DNP	Gas	10 s	64%/46%	5	200 °C	1000 cycles	This work

Table S3. The performance of 2DP$_{TAPB+TPAK}$ in the detection experiment of metal ion.

Material	Status	Metal ion	Responding speed	K_{sv} (M^{-1})	LOD (M)	Recovery rate	Cycles	High temperature resistance	Bending resistance	Literature
COF-JLU3	Powder	Cu^{2+}	30 min	3.8×10^{4}	3.1×10^{-7}	-	-	-	-	9
COF-LZU8	Powder	Hg^{2+}	immediately	-	2.5×10^{-8}	-	3	-	-	10
NUS-24	Nanosheets	Fe^{3+}	5 min	2.7×10^{4}	9.0×10^{-4}	-	-	-	-	8
DhaTab	Powder	Fe^{3+}	1 min	1.0×10^{5}	1.2×10^{-7}	-	-	-	-	11
Bth-Dma	Powder	Fe^{3+}	immediately	2.3×10^{4}	1.7×10^{-7}	-	-	-	-	12

$2DP_{TPAK+TAPB}$	Film	Fe^{3+}	60 s	2.7×10^{5}	4.2×10^{-7}	98.72-106.89%	5	200 °C	1000 cycles	This work

References

1. Dalapati, S.; Jin, S.; Gao, J.; Xu, Y. H.; Nagai, A. Jiang, D. L. An Azine-linked Covalent Organic Framework. *J. Am. Chem. Soc.* **2013**, 135, 17310–17313.
2. Das, G.; Biswal, B. P.; Kandambeth, S.; Venkatesh, V.; Kaur, G.; Addicoat, M.; Heine, T.; Verma, S.; Banerjeee, R. Chemical sensing in two dimensional porous covalent organic nanosheets. *Chem. Sci.* **2015**, 6, 3931–3939.
3. Lin, G. Q.; Ding, H. M.; Yuan, D. Q.; Wang, B. S.; Wang, C. A pyrene-based, fluorescent three-dimensional covalent organic framework. *J. Am. Chem. Soc.* **2016**, 138, 3302–3305.
4. Zhang, C. L.; Shi, M. Highly fluorescent polyimide covalent organic nanosheets as sensing probes for the detection of 2,4,6-trinitrophenol. *ACS Appl. Mater. Inter.* **2017**, 9, 13415–13421.
5. Zhu, M. W.; Xu, S. Q.; Wang, X. Z.; Chen, Y. Q.; Dai, L. Y.; Zhao, X. The construction of fluorescent heteropore covalent organic frameworks and their applications in spectroscopic and visual detection of trinitrophenol with high selectivity and sensitivity. *Chem. Commun.* **2018**, 54, 2308–2311.
6. Jiang, S.; Liu, S. D.; Meng, L. C. Covalent organic hollow nanospheres constructed by using AIE active units for nitrophenol explosives detection. *Sci. China. Chem.* **2020**, 063(004), 497–503.
7. Dong, J. Q.; Tummanapelli, A. K.; Li, X.; Ying, S. M.; Hirao, H.; Zhao, D. Fluorescent porous organic frameworks containing molecular rotors for size-selective recognition. *Chem. Mater.* **2016**, 28, 7889–7897.
8. Dong, J.; Zhang, K.; Li, X.; Ultrathin two-dimensional porous organic nanosheets with molecular rotors for chemical sensing. *Nat. Commun.* **2017**, 8, 1142–1156.
9. Li, Z.; Zhang, Y.; Xia, H.; Ying, M.; Liu, X. M. A robust and luminescent covalent organic framework as a highly sensitive and selective sensor for the detection of Cu^{2+} ions. *Chem. Commun.* **2016**, 52, 6613–6616.
10. Ding, S. Y.; Dong, M.; Wang, Y. W.; Chen, Y. T.; Wang, H. Z.; Su, C. Y.; Wang, W. Thioether-based fluorescent covalent organic framework for selective detection and facile removal of mercury(II). *J. Am. Chem. Soc.* **2016**, 138, 3031–3037.
11. Wang, L. L.; Yang, C. X.; Yan, X. P. Exploring fluorescent covalent organic frameworks for selective sensing of Fe^{3+}. *Sci. China. Chem.* **2018**, 61, 1470–1474.
12. Chen, G.; Lan, H. H.; Cai, S. L.; Sun, B.; Li, X. L.; He, Z. H.; Zheng, S. R.; Fan, J.; Liu, Y.; Zhang, W. G. Stable hydrazone-linked covalent organic frameworks containing O,N,O′-chelating sites for Fe(III) detection in water. *ACS Appl. Mater. Inter.* **2019**, 11, 12830–12837.
